# Evidence for binary Smc complexes lacking kite subunits in archaea

**DOI:** 10.1107/S2052252519016634

**Published:** 2020-01-16

**Authors:** Jae-Hyun Jeon, Han-Sol Lee, Ho-Chul Shin, Mi-Jeong Kwak, Yeon-Gil Kim, Stephan Gruber, Byung-Ha Oh

**Affiliations:** a Department of Biological Science, KAIST Institute for the Biocentury, Korea Advanced Institute of Science and Technology, Daejeon 34141, Republic of Korea; b Disease Target Structure Research Center, Korea Research Institute of Bioscience and Biotechnology, Daejeon 34141, Republic of Korea; c CKD Research Institute, ChongKunDang Pharmaceutical Corp., Yongin, Gyeonggi 16995, Republic of Korea; d Pohang Accelerator Laboratory, Pohang University of Science and Technology, Pohang, Kyungbuk 37673, Republic of Korea; e Department of Fundamental Microbiology, University of Lausanne, Bâtiment Biophore, 1015 Lausanne, Switzerland

**Keywords:** Smc, ScpA, ScpB, archaea, kite proteins, Smc–ScpA, Smc–ScpAB, condensin

## Abstract

SMC complexes play a central role in chromosome organization in all domains of life. The bacterial Smc–ScpAB complex is a three-subunit complex composed of Smc, ScpA and ScpB. ScpA bridges the two ATPase domains of the Smc homodimer, while ScpB, which belongs to the kite family of proteins, interacts with ScpA. The three subunits are known to be equally important for the function of Smc–ScpAB in bacteria. From crystallographic and biochemical studies, evidence is provided that six archaeal ScpA proteins are unable to interact with the only putative ScpB found in these species. Structure-based sequence alignment reveals that these archaeal ScpAs lack the ScpB-binding segment that is commonly present in the middle of bacterial ScpA sequences, which is thus responsible for their inability to interact with ScpB. ScpA proteins lacking the ScpB-binding segment are found to prevail in archaea. Moreover, two archaeal ScpA proteins with a longer middle region also failed to bind their putative ScpB partner. Furthermore, all or most species belonging to five out of 14 euryarchaeotal orders contain Smc and ScpA but not a detectable ScpB homologue. These data support the notion that archaeal Smc-based complexes generally function as a two-subunit complex composed of only Smc and ScpA.

## Abstract 2

Contrary to the general concept, the archaeal Smc-based complex appears to lack the kite subunit ScpB that is essential in the bacterial Smc–ScpAB complex that mediates chromosome organization.

## 1. Introduction   

During cell division, faithful chromosome segregation and partitioning into two daughter cells relies on large protein complexes called condensins in eukaryotes or condensin-like complexes in prokaryotes (Kamada *et al.*, 2017 ▸; Nasmyth & Haering, 2005 ▸). All condensin complexes commonly contain two subunits belonging to the family of SMC (structural maintenance of chromosomes) proteins, which form a homodimer or heterodimer. The SMC proteins contain a hinge domain and an ATPase domain that are separated by an ∼50 nm long coiled-coil arm. The hinge domain serves as the dimerization interface, and the ATPase domain undergoes an engagement and disengagement cycle. In this cycle, ATP binding induces the engagement of two ATPase domains, and subsequent ATP hydrolysis results in their disengagement. In addition to the SMC subunits, prokaryotic condensin-like complexes typically have two non-SMC subunits, whereas eukaryotic condensins have three non-SMC subunits (Uhlmann, 2016 ▸). Three different types of condensin-like complexes have been discovered in bacteria. One is the Smc–ScpAB complex, which is composed of an Smc homodimer and two non-SMC subunits, ScpA and ScpB, that form a tight binary complex (usually referred to as ScpAB). This condensin-like complex is also found in archaea (Soppa *et al.*, 2002 ▸; Mascarenhas *et al.*, 2002 ▸; Barillà, 2016 ▸; Kamada & Barillà, 2018 ▸). The other two are MukBEF and MksBEF, which are composed of the SMC subunit MukB (or MksB) and two non-SMC proteins called MukE and MukF (or MksE and MksF) that also form a binary complex (Niki *et al.*, 1991 ▸; Yamanaka *et al.*, 1996 ▸; Yamazoe *et al.*, 1999 ▸; Petrushenko *et al.*, 2011 ▸). Smc–ScpAB is more closely related to the eukaryotic condensins and is much more widely spread in bacteria than MukBEF and MksBEF (Cobbe & Heck, 2004 ▸). In Smc–ScpAB, ScpA is a kleisin subunit that binds two distinct interfaces of Smc: one on the coiled-coil segment close to the ATPase domain (dubbed the neck) and the other at the bottom of the ATPase domain (dubbed the cap) (Bürmann *et al.*, 2013 ▸). Binding on the neck and on the cap are through the N-terminal α-helical domain (NαHD) and the C-terminal winged-helix domain (cWHD) of ScpA, respectively. These interactions result in an asymmetric 2:1 complex between Smc and ScpA, despite two molecules of Smc forming a symmetric homodimer (Bürmann *et al.*, 2013 ▸).

The bacterial ScpB and MukE proteins (and the eukaryotic Nse1 and Nse3 proteins in the Smc5/6 complex) comprise tandem WHDs and are classified into a new family of proteins called kite (kleisin-interacting tandem winged-helix elements of SMC complexes) proteins (Palecek & Gruber, 2015 ▸). In the structurally characterized ScpAB complexes derived from *Streptococcus pneumoniae* and *Geobacillus stearothermo­philus*, two molecules of ScpB bind to the middle region of ScpA between the NαHD and the cWHD (Kamada *et al.*, 2013 ▸; Bürmann *et al.*, 2013 ▸). This middle region is a linear segment that adopts a ‘rope-like’ shape, and thus would be flexible by itself. The binding of two ScpB molecules renders the middle segment conformationally rigid and physically separates the NαHD from the cWHD by about 40 Å, indicating that one functional role of ScpB lies in shaping the central ScpA structure.

Disruption of ScpB is known to be as detrimental as null mutation of Smc or ScpA in *Bacillus subtilis* (Mascarenhas *et al.*, 2002 ▸), implying that the kite subunit is an integral component of Smc–ScpAB. However, prokaryotes which harbor Smc and ScpA apparently do not always harbor ScpB (Soppa *et al.*, 2002 ▸). In particular, some euryarchaeotal species lack obvious ScpB homologues in their genome, suggesting the existence of an Smc-based complex that functions without a kite subunit.

We purified six pairs of archaeal ScpA and ScpB homologues and investigated their intermolecular interactions to find strong evidence for a lack of direct association. Based on the structural characterization of *Thermococcus onnurineus* ScpA and the putative *Pyrococcus yayanosii* ScpB, the lack of interaction is ascribed to the absence of a sequence that corresponds to the ScpB-binding interface in bacterial ScpAs. Genome-wide sequence analyses reveal that the middle region of archaeal ScpAs is generally significantly shorter than that of bacterial ScpAs, and that a ScpB homologue is apparently absent from a range of archaeal species. We show that the N-terminal domain of *T. onnurineus* ScpA (*To*ScpA^N^), however, interacts with the Smc neck but only weakly, which has similarly been observed in a number of bacterial Smc–ScpAB complexes. Thus, a large fraction of archaeal Smc-based complexes appear to function as a binary complex between Smc and ScpA.

## 2. Methods   

### 2.1. Protein and gene-locus search   

The existence and gene loci of Smc, ScpA and putative ScpB proteins were searched for using a Python script (provided as supporting information) and a *BLAST* search. Each order in the phylum Euryarchaeota and other phyla from the archaea was searched in the NCBI Assembly database (https://www.ncbi.nlm.nih.gov/assembly/), and the reference sequence (RefSeq) data were downloaded in genomic general feature format (GFF). The existence of *smc*, *scpA* and *scpB* genes was determined by searching for the strings ‘chromosome segregation protein SMC’, ‘segregation/condensation protein A’ or ‘ScpA’ and ‘segregation and condensation protein B’ or ‘ScpB’, respectively, in each GFF file. The output files created by the script were composed of three parts: Results, Analyses and Outliers. The Results part contains the taxonomic identification, locus tags and product names of the genes flanking *scpA*. The Analyses part contains the number of species containing *smc*, *scpA* or *scpB* genes, the number of species in which *smc* and *scpA* neighbor each other, the number of species in which *scpA* and *scpB* neighbor each other, and the numbers of each of the three genes that are marked as a ‘pseudogene’. The Outliers part contains taxonomic identifications of species in which any of the three genes is not found in the GFF file.

If any one of the three genes was not found in a GFF file, a *BLAST* search was performed in two steps to clarify whether the gene(s) is (are) actually missing or present but annotated with other names such as ‘hypothetical protein’. In the first step, *T. onnurineus* Smc, ScpA or ScpB was used to identify the closest homologues in each of the archaeal orders or phyla, which are listed in Supplementary Table S1. In the second step, one of these proteins was selected as a query for each *BLAST* search against a specific genome where one of the three genes is undetected in the string searches. In the *BLAST* searches, proteins exhibiting higher than 20% sequence identity with greater than 70% coverage of the query sequence were counted as Smc, ScpA or ScpB. Additionally, proteins exhibiting higher than 50% identity with greater than 25% coverage were counted as ScpA, since only the N-terminus of the query was often aligned.

Independently, an HMM search was performed against the ‘nr_arc_1_Oct’ database using the sequence of Smc, ScpA or ScpB from *T. onnurineus* as a query. Sequences exhibiting *E*-values of less than 1.0 × 10^−4^, 1.0 × 10^−4^ or 1.0 × 10^−100^ were counted as ScpA, ScpB or Smc, respectively. These *E*-values were chosen as sequences with a higher *E*-value are often annotated as an unrelated protein. The presence or absence of the three proteins was compared using the *BLAST* search for the source archaeal organisms in the RefSeq database.

### 2.2. Protein production   

All of the protein constructs used in this study are listed in Supplementary Table S2. Each construct was cloned and transformed into the *Escherichia coli* BL21 (DE3) RIPL strain. The cells were grown in Luria–Bertani medium and the proteins were expressed at 18°C for 18 h after induction with 0.1 m*M* isopropyl β-d-1-thiogalactopyranoside.

For all of the proteins that contain a CPD (cysteinyl protease domain)-(His)_10_ tag (Shen *et al.*, 2009 ▸), the cells were harvested and sonicated in a buffer solution (buffer *A*) consisting of 20 m*M* Tris–HCl pH 7.5, 100 m*M* NaCl, 3 m*M* β-mercaptoethanol (β-ME) and the supernatant was applied onto a column filled with HisPur cobalt resin (Thermo Scientific). The resin was washed with buffer *A* containing an additional 10 m*M* imidazole. The CPD-(His)_10_ tag was removed by on-gel digestion with 0.1 m*M* phytate, which activates CPD. The proteins were further purified using a HiTrap Q anion-exchange column (GE Healthcare) and a HiLoad 26/60 Superdex 75 gel-filtration column (GE Healthcare) in buffer *A*.

For the purification of proteins containing a GFP-(His)_10_, (His)_10_-GST or (His)_10_-MBP tag, the cell lysates were obtained in buffer *A* and the supernatant was applied onto the cobalt resin. The proteins were eluted from the resin using buffer *A* containing an additional 150 m*M* imidazole. If necessary, the eluted proteins were treated with Tobacco etch virus protease at 4°C for 4 h to cleave the tag, dialyzed against buffer *A* and applied onto the cobalt resin again to remove the tag. The proteins were further purified using a HiTrap Q anion-exchange column and a HiLoad 26/60 Superdex 75 gel-filtration column in buffer *A*. All of the purified proteins in buffer *A* were concentrated, flash-frozen in liquid nitrogen and stored at −80°C.

For co-expression experiments, vectors encoding *P. yayanosii* ScpA (*Py*ScpA) with an N-terminal (His)_10_-MBP tag and PYCH_12850 (the putative *P. yayanosii* ScpB) without a tag were introduced into the *E. coli* BL21 (DE3) RIPL strain. The proteins were expressed as described above. The cell lysate was obtained in buffer *A* and the supernatant was applied onto the cobalt resin. The proteins were eluted from the resin using buffer *A* containing an additional 150 m*M* imidazole. The eluents were subjected to denaturing polyacrylamide gel electrophoresis (PAGE) to determine whether the two proteins eluted together or separately.

### 2.3. Crystallization, data collection and structure determination   

Selenomethionine (SeMet)-substituted *To*ScpA^N^ and PYCH_12850 were expressed in the *E. coli* B834 (DE3) RIL strain (Novagen) and purified as described above. SeMet-substituted *To*ScpA^N^ (at 4.5 mg ml^−1^ in buffer *A*) was crystallized using a solution consisting of 3.5 *M* sodium formate, 0.1 *M* sodium malonate. The crystals were grown at 22°C in sitting drops consisting of 1.5 µl protein solution and 1.5 µl reservoir solution. The crystals were dehydrated by adding 30 µl reservoir solution consisting of 3.5 *M* sodium formate, 0.1 *M* sodium malonate, 10% glycerol to the crystal-containing drops followed by air exposure for 2 h at 22°C (Heras & Martin, 2005 ▸). SeMet-substituted PYCH_12850 (82.1 mg ml^−1^ in buffer *A*) was crystallized in a solution consisting of 35%(*v*/*v*) pentaerythritol propoxylate (5/4 PO/OH, Hampton Research), 0.1 *M* MES pH 5.5, 0.4 *M* sodium chloride. The crystals were grown at 22°C in hanging drops consisting of 1.5 µl protein solution and 1.5 µl reservoir solution. Single-wavelength anomalous dispersion (SAD) data sets for *To*ScpA^N^ and PYCH_12850 were collected on beamlines 5C and 11C at Pohang Accelerator Laboratory, Republic of Korea. The SAD data set for *To*ScpA^N^ was processed using the *HKL*-2000 suite (Otwinowski & Minor, 1997 ▸). Four SAD data sets for PYCH_12850 were integrated using *MOSFLM* (Battye *et al.*, 2011 ▸) and were merged and scaled using *BLEND* (Foadi *et al.*, 2013 ▸; Evans & Murshudov, 2013 ▸; Evans, 2011 ▸), which are included in the *CCP*4 suite (Winn *et al.*, 2011 ▸). Phasing and initial model building were performed using *AutoSol* in *Phenix* (Terwilliger *et al.*, 2009 ▸). Manual model building and structure refinement were performed using *Coot* (Emsley *et al.*, 2010 ▸), *CNS* (Brünger *et al.*, 1998 ▸) and *Phenix* (Afonine *et al.*, 2012 ▸; Liebschner *et al.*, 2019 ▸). Crystallographic data statistics for the two SeMet-substituted proteins are summarized in Table 1 ▸.

### 2.4. Pull-down assay   

For (His)_10_ pull-down assays, (His)_10_-MBP-tagged ScpAs and untagged putative ScpBs were used. The pairs of proteins (1 µ*M* each) were incubated in 100 µl buffer solution consisting of 20 m*M* Tris–HCl pH 7.5, 100 m*M* NaCl at 25°C for 10 min. The reaction mixtures were then incubated with 15 µl Ni–NTA resin (Thermo). The resin was washed three times with buffer consisting of 20 m*M* Tris–HCl pH 7.5, 100 m*M* NaCl, 30 m*M* imidazole and subjected to denaturing PAGE to determine whether untagged putative ScpB was retained on the resin together with tagged ScpA.

### 2.5. Size-exclusion chromatography   

Size-exclusion chromatography was performed using a Superdex 200 Increase 10/300 GL column (GE Healthcare) in a running buffer consisting of 20 m*M* Tris–HCl pH 7.5, 100 m*M* NaCl, 1 m*M* DTT at 4°C. Each protein or protein mixture (10 µ*M* each in 100 µl) was injected into the column.

### 2.6. Bismaleimidoethane (BMOE)-mediated cysteine cross-linking   

Four constructs (wild type, Q185C, Q994C and A1110C) of the *T. onnurineus* Smc (*To*Smc) head domain with an 80-residue coiled-coil stretch (*To*SmcHd-CC80), which were co-purified with *To*ScpA(E69C)-GFP-(His)_10_, were dialyzed in a buffer solution consisting of 20 m*M* Tris–HCl pH 7.5, 100 m*M* NaCl. BMOE (Thermo Scientific) was then added to a final concentration of 200 µ*M* to each mixture at 2 µ*M*. The reaction mixtures were incubated for 10 min at 25°C, quenched by adding β-ME (14 m*M*) and subjected to denaturing PAGE. The protein bands were visualized by both GFP signal and Coomassie Blue staining.

## 3. Results   

### 3.1. Absence or incomplete presence of *smc*, *scpA* and *scpB* in archaeal genomes   

**Figure 1:**
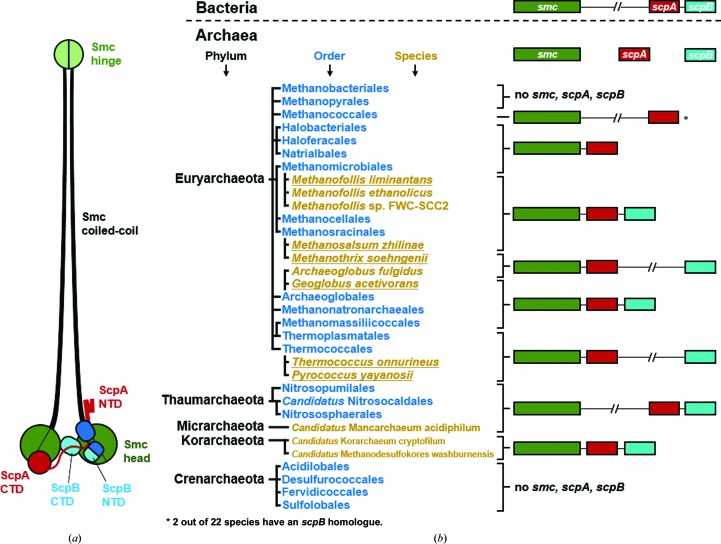
The co-occurrence of *smc*, *scpA* and *scpB* across prokaryotes. (*a*) A schematic drawing of the bacterial Smc–ScpAB complex is shown on the left. (*b*) Phyla, orders and selected species are shown and color-coded. All of the orders in Archaea are shown if at least one RefSeq assembly is available. Species are shown if they were the sources of the proteins used in this study (underlined), if the genomic loci of *smc*, *scpA* and *scpB* vary in the same order and if an order is undefined.

We performed a genome-wide search for the presence of *smc*, *scpA* and *scpB* and their co-occurrence in archaeal organisms using a Python script (see Section 2), the STRING database (http://string-db.org/; Szklarczyk *et al.*, 2015 ▸), *BLAST* (Mount, 2007 ▸) and HMM (Zimmermann *et al.*, 2018 ▸) searches. The *BLAST* search was restricted to organisms for which the species has been identified and the genome sequence is registered in the NCBI RefSeq database (a total of 640 genomes), and the HMM search was compared for these organisms. Our search did not detect *smc*, *scpA* and *scpB* genes in the archaeal phylum Crenarchaeota and in the classes Methanobacteria and Methanopyri of the phylum Euryarchaeota [Fig. 1 ▸(*b*) and Table 2 ▸], suggesting that these organisms are unlikely to encode an Smc-based complex. Notably, *Sulfolobus* species belonging to the Crenarchaeota branch have recently been found to utilize a novel SMC-like protein called coalescin for chromosome organization (Takemata *et al.*, 2019 ▸). Members of the other branches of Euryarchaeota have both *smc* and *scpA*. These two genes are next to each other on the genome in most of these euryarchaeotal species (433 out of 469 species) and in the species from Korarchaeota (three species). The two genes, however, are separated from each other in all Thaumarchaeota species (14 species), in all of the species belonging to the order Methanococcales (22 species), in three out of 62 species belonging to the order Methanosarcinales and in 11 out of 44 species in the order Thermococcales [Fig. 1 ▸(*b*) and Table 2 ▸].

An obvious *scpB* gene is not always found on the archaeal genomes where *smc* and *scpA* are found: it was not detected in any of the species belonging to the class Halobacteria (285 species) and most of the species belonging to the orders Methanomicrobiales (25 out of 28 species) and Methanococcales (20 out of 22 species) [Fig. 1 ▸(*b*) and Table 2 ▸]. In terms of number of species, 480 out of the total of 640 analyzed archaeal genomes do not contain a readily detectable *scpB* homologue. The result of the HMM search was basically identical to that of the *BLAST* search, uncovering no additional credible homologues (Table 2 ▸). Of note, in the orders Methanococcales, Thermoplasmatales and Thermococcales, where *scpB* is found, this gene is remotely located from *scpA* in all of the member species, in contrast to the juxtaposition of *scpA* and *scpB* in most bacterial genomes, as noted previously (Kamada & Barillà, 2018 ▸). In a total of 61 out of 160 archaeal species containing both *scpA* and *scpB*, *scpB* is remotely located from *scpA* in the genome, and this is observed in three out of nine euryarchaeotal orders [Fig. 1 ▸(*b*) and Table 2 ▸].

The absence of *scpB* in a range of euryarchaeotal orders indicates that the Smc-based complex in these organisms does not require the kite subunit ScpB. The separated positioning of *scpB* from *scpA* in many Euryarchaeota species highlights the possibility that at least some genes annotated as *scpB* in these organisms may encode functionally unrelated paralogues, rather than true orthologues, of ScpB.

### 3.2. Absence of interaction between ScpA and ScpB derived from euryarchaeal branches   

**Figure 2:**
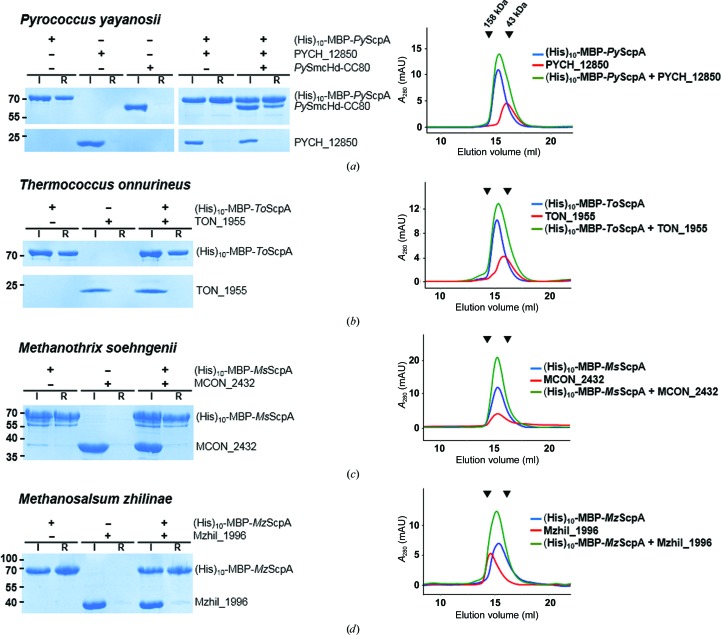
Pull-down assay and size-exclusion chromatography for pairs of archaeal ScpA and ScpB homologues. The name of the organism from which the two proteins were derived is shown at the top of each panel. Denaturing gels and chromatograms are shown on the left and right, respectively. (His)_10_-MBP-tagged ScpA proteins were mixed with the indicated ScpB homologues (TON_1955, PYCH_12850, MCON_2432 and Mzhil_1996) in a 1:1 molar ratio (1 µ*M* each). ‘I’ and ‘R’ stand for the input proteins loaded onto the Ni–NTA resin and the resin-bound proteins, respectively. Molecular weights are labelled in kDa. For chromatography, individual proteins or their mixture (100 µg each) were loaded onto an analytical gel-filtration column. The two black triangles at the top of the chromatograms indicate the elution positions of the molecular-weight markers: 158 kDa (left) and 43 kDa (right).

In the order Thermococcales, to which the genera *Pyrococcus* and *Themococcus* belong, *smc* and *scpA* are present according to our genomic analysis (Table 2 ▸). Three quarters of the analyzed gene pairs are next to each other, while the rest of them are found to be separated from each other by two intervening genes. Remotely from *smc* and *scpA*, *scpB* appears to also be present in these organisms [Fig. 1 ▸(*b*)]. In the *P. yayanosii* proteome, PYCH_01210 and PYCH_12850 are the sole plausible homologues of ScpA and ScpB, respectively. We first tested the potential interaction between PYCH_01210 (denoted *Py*ScpA) and PYCH_12850. (His)_10_-MBP-tagged *Py*ScpA and PYCH_12850 without a tag were purified separately, and the two proteins were subjected to a (His)_10_ pull-down assay in the presence of untagged *P. yayanosii* Smc (*Py*Smc) head domain with an 80-residue coiled-coil stretch (*Py*SmcHd-CC80). The three proteins were incubated together, loaded onto immobilized Ni^2+^ resin and resin-bound proteins were visualized on a denaturing polyacrylamide gel. In this experiment untagged PYCH_12850 was not observed in the eluate, indicating that the protein did not interact with (His)_10_-MBP-tagged *Py*ScpA and thus was not retained on the resin [Fig. 2 ▸(*a*)]. In contrast, a protein band corresponding to *Py*SmcHd-CC80 was clearly observed, which is consistent with the known interactions between ScpA and the Smc head domain [Fig. 2 ▸(*a*)]. The mixture of (His)_10_-MBP-tagged *Py*ScpA and PYCH_12850 was subjected to size-exclusion chromatography. No elution peak corresponding to complex formation was observed, also indicating that the proteins do not interact with each other. Co-expression of the two proteins in *E. coli* also did not result in complex formation between the two proteins (not shown), which is in sharp contrast to bacterial ScpA–ScpB pairs, which can readily be co-purified (Kamada *et al.*, 2017 ▸; Bürmann *et al.*, 2013 ▸).

Likewise, ScpA from *T. onnurineus* (TON_1071; denoted *To*ScpA) and the putative ScpB from this organism (TON_1955) did not exhibit a detectable interaction with each other [Fig. 2 ▸(*b*)]. The data obtained for the purified recombinant proteins are consistent with the fractionation of the native proteome of *P. furiosus*, showing that peptides derived from PF1842 (ScpA) and PF1843 (Smc) were detected in the same fraction, while those of PF2021 (ScpB) were detected in different fractions (Menon *et al.*, 2009 ▸; F. Poole, personal communication).

We also tested the potential interaction between ScpA and the putative ScpB protein from *Methanosalsum zhilinae* and *Methanothrix soehngenii*, which are evolutionarily distant, from the order Thermococcales [Fig. 1 ▸(*b*)]. Similar (His)_10_ pull-down assays exhibited no significant interaction between these two pairs [Figs. 2 ▸(*c*) and 2 ▸(*d*)]. Of note, the *scpA* and *scpB* genes of *M. zhilinae* are direct neighbors, like those in bacterial genomes, while those of *M. soehngenii* are distantly located in the genome [Fig. 1 ▸(*b*)]. Thus, the lack of interaction is unrelated to the genomic loci of *scpA* and *scpB*.

### 3.3. Structure of the ScpB homologue from *P. yayanosii*   

**Figure 3:**
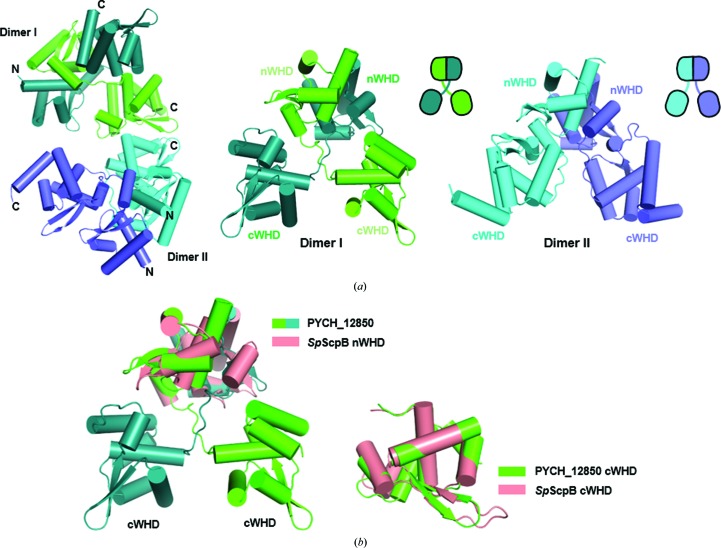

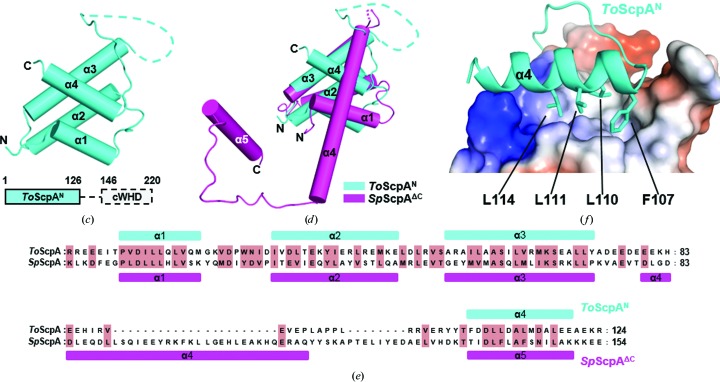
Structures of the ScpB homologue PYCH_12850 and *To*ScpA^N^. (*a*, *b*) Structural features of PYCH_12850. (*a*) Two independent dimers in the crystal (left). The protein forms a homodimer through the N-terminal WHD (nWHD), which is followed by a flexible linker segment and a dangling cWHD (middle and right). The relative positions of the two domains differ in the two dimers. (*b*) Superposition of PYCH_12850 onto *S. pneumoniae* ScpB. The nWHDs and cWHDs are separately superposed. The nWHD–nWHD interaction as well as the structures are closely similar. (*c*)–(*f*) Structural features of *To*ScpA^N^. (*c*) Overall structure. The structure is composed of four stacked α-helices. Residues 75–94 are disordered and indicated by a dotted line. Shown below is a representation of full-length *To*ScpA. The C-terminal region forms the cWHD. (*d*) Structural alignment onto *Sp*ScpA^ΔC^. The α-helices are labeled in their order of appearance in the structures. The structures are closely similar but only up to α3. (*e*) Sequence alignment of *To*ScpA^N^ and *Sp*ScpA^ΔC^. Reflecting the structural difference shown in (*d*), the sequence between α3 and α4 of *To*ScpA^N^ is notably different from that between α3 and α5 of *Sp*ScpA^ΔC^. (*f*) Intramolecular hydrophobic interaction of α4 with the rest of the protein. The interacting hydrophobic residues on α4 are shown as sticks and the rest of the protein is shown as an electrostatic surface potential map.

To understand the structural features that may hinder interaction between archaeal ScpA proteins and putative archaeal ScpB proteins, we next determined the crystal structure of full-length PYCH_12850 at 3.0 Å resolution [Fig. 3 ▸(*a*)]. The protein is composed of N- and C-terminal WHDs (nWHD and cWHD, respectively) that are connected by a long intervening loop. This loop appears to be flexible, because the two molecules of PYCH_12850 in the asymmetric units show that the orientation of the cWHD relative to the nWHD in one molecule is quite different from that in the other molecule [Fig. 3 ▸(*a*)]. PYCH_12850 forms a homodimer through the interaction between the nWHDs [Fig. 3 ▸(*a*)]. These structural features have been commonly observed in the structures of bacterial ScpB proteins (Kamada *et al.*, 2013 ▸; Bürmann *et al.*, 2013 ▸). Structural superposition of *S. pneumoniae* ScpB (bound to ScpA; Bürmann *et al.*, 2013 ▸) and PYCH_12850 showed that the N-terminal domains of the two structures are similar to each other and so are the C-terminal domains [Fig. 3 ▸(*b*)], as expected from the ∼48% sequence similarity between the two proteins. We could not find a particular structural feature that may prevent the archaeal sequence homologue of ScpB from interacting with ScpA, indicating that features of archaeal ScpA may prevent interaction with ScpB.

### 3.4. Structure of an N-terminal fragment of *T. onnurineus* ScpA   

We next determined the crystal structure of a *T. onnurineus* ScpA fragment composed of residues 1–126, referred to as *To*ScpA^N^, at 2.5 Å resolution. This construct lacks the cWHD and the preceding linker segment (residues 127–220). *To*ScpA^N^ folds into an all-α-helical tertiary structure containing four α-helices and connecting loops [Fig. 3 ▸(*c*)]. In comparison with the structure of *S. pneumoniae* ScpA lacking the cWHD (*Sp*ScpA^ΔC^) in complex with ScpB (*Sp*ScpB; Bürmann *et al.*, 2013 ▸), *To*ScpA^N^ is similar to *Sp*ScpA^ΔC^ up to the third α-helix [Fig. 3 ▸(*d*)]. In contrast, the fourth α-helix (α4) of *To*ScpA^N^ and the preceding loop appear to be unrelated to any part of *Sp*ScpA^ΔC^. According to a multiple sequence alignment using *HHpred* (Söding *et al.*, 2005 ▸), α4 of *To*ScpA^N^ (residues 107–121) corresponds to α5 of *Sp*ScpA^ΔC^ (residues 137–151) [Fig. 3 ▸(*e*)]. In the structural alignment, these two α-helices are far from each other, indicating that the fourth α-helix of *To*ScpA^N^ has to be detached from the α1–α3 bundle in order to occupy the same spatial position as α5 of *Sp*ScpA^ΔC^. Such a separation is unlikely to happen because α4 is tightly packed against α1–α3 via hydrophobic interactions [Fig. 3 ▸(*f*)] and, consistently, a construct lacking α4 was expressed as an insoluble form in *E. coli* (not shown). Intriguingly, however, the possibility of α4 detachment in the functional cycle of the holo complex cannot be ruled out. The structure thus highlights the different organization in the middle region of a bacterial and an archaeal ScpA protein.

### 3.5. Archaeal ScpAs generally lack the ScpB-binding interface   

**Figure 4:**
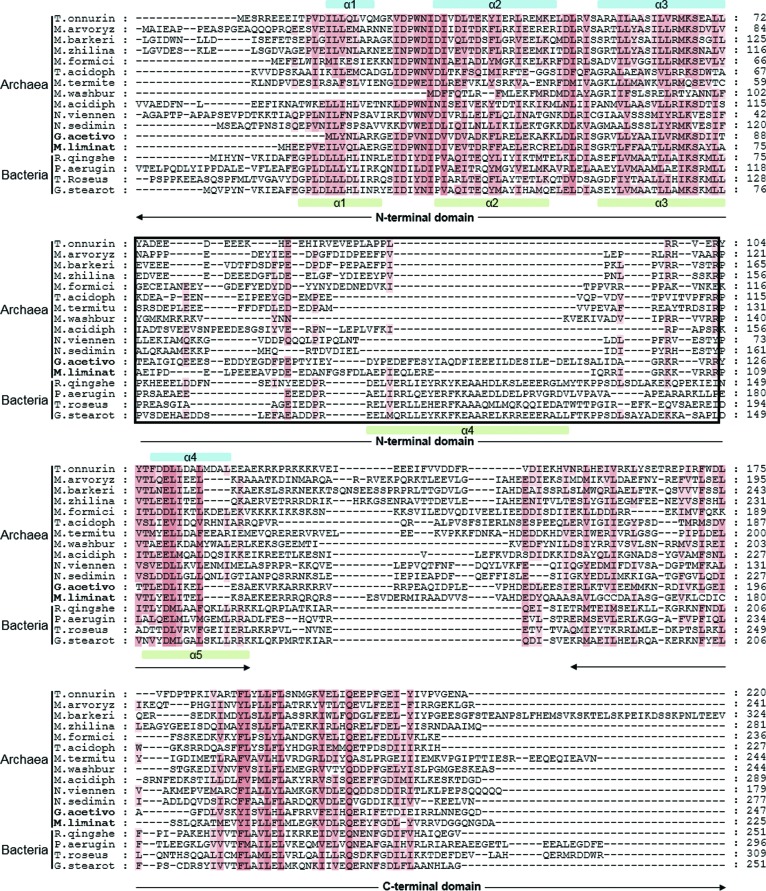
Multiple sequence alignment of ScpAs from archaea and bacteria. ScpA sequences derived from nine archaeal species and four bacterial species were aligned using *Clustal Omega* (Sievers *et al.*, 2011 ▸). Secondary-structural elements of *To*ScpA^N^ and *Gs*ScpA^ΔC^ are indicated at the top and the bottom of the alignment, respectively. The black box indicates the region of ScpA^N^ that shows a clear difference between archaeal and bacterial ScpA proteins. T.onnurin, *Thermococcus onnurineus* (gi:212009177); M.arvoryz, *Methanocella arvoryzae* (gi:500971514); M.barkeri, *Methanosarcina barkeri* (gi:805410469); M.zhilina, *Methanosalsum zhilinae* (gi:335931289); M.liminat, *Methanofollis liminatans* (gi:395441815); M.formici, *Methanotorris formicicus* (gi:373561535); G.acetivo, *Geoglobus acetivorans* (gi:728876205); T.acidoph, *Thermoplasma acidophilum* (gi:851298378); M.termitu, *Candidatus* Methanoplasma termitum (gi 851220379); M.washbur, *Candidatus* Methanodesulfokores washburnensis (gi:1538762872); M.acidiph, *Candidatus* Mancarchaeum acidiphilum (gi:1214173397); N.viennen, *Nitrososphaera viennensis* (gi:647811207); N.sedimin, *Candidatus* Nitrosopumilus sediminis (gi:407047581); R.qingshe, *Rhodococcus qingshengii* (gi:1595910292); P.aerugin, *Pseudomonas aeruginosa* (gi:1440714951); T.roseus, *Terriglobus roseus* (gi:1124365071); G.stearot, *Geobacillus stearothermophilus* (gi:1017231538). For expanded and grouped sequence alignments, see Supplementary Figs. S1 and S2.

A multiple sequence alignment of ScpAs from phylo­genetically remote archaeal species shows that archaeal ScpAs are homologous to bacterial ScpAs in the N- and C-terminal regions (Fig. 4 ▸). In contrast, the middle region in the archaeal ScpAs, corresponding to the segment between α3 and α4 in the *To*ScpA^N^ structure, is clearly different from that in the bacterial ScpAs, corresponding to the segment between α3 and α5 in the *G. stearothermophilus* ScpA^ΔC^ (*Gs*ScpA^ΔC^) structure (Kamada *et al.*, 2013 ▸). This middle region in most archaeal species is notably shorter than that in bacterial ScpAs and exhibits virtually no significant sequence homology throughout archaeal species (black boxes in Fig. 4 ▸ and Supplementary Figs. S1 and S2). In the archaeal species shown in the alignment, the number of amino acids in the middle region in archaeal ScpAs varies from 30 to 66, while that in bacterial ScpAs varies from 61 to 72 (black box in Fig. 4 ▸; Table 3 ▸). Critically, the crystal structures of ScpA^ΔC^–ScpB complexes (PDB entries 4i98 and 3w6j) show that this middle region in bacterial ScpA contains the major ScpB-binding interface in the second half of α4 (Kamada *et al.*, 2013 ▸; Bürmann *et al.*, 2013 ▸). These observations provide a plausible explanation for why ScpAs derived from the four archaeal species failed to interact with the putative ScpB homologues (Fig. 2 ▸).

**Figure 5:**
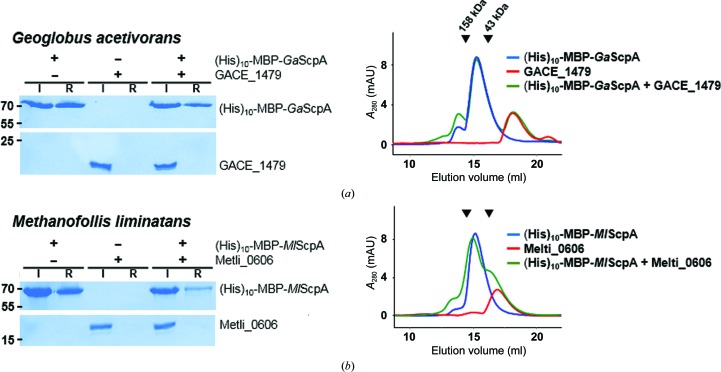
Protein-binding assay for archaeal ScpAs with a longer middle region. The indicated ScpA and ScpB homologue were mixed together in a 1:1 molar ratio (1 µ*M* each), loaded onto the resin and analyzed by denaturing gel electrophoresis (left) or on an analytical size-exclusion column (right). The name of the source organism is shown at the top. Molecular weights are labelled in kDa for the gels. The two black arrows indicate the elution positions of the molecular-weight markers. GACE_1479 (174 amino acids) appears as a monomeric protein and Metli_0606 (161 amino acids) as a smaller dimeric protein in comparison with the ScpB homologues in Fig. 2 ▸ (192–364 amino acids).

Some archaeal ScpAs have a longer middle region. For example, *Geoglobus acetivorans* ScpA (*Ga*ScpA) and *Methano­follis liminatans* ScpA (*Ml*ScpA) have 66- and 49-residue-long middle regions, respectively (bold letters in Fig. 4 ▸). While the two proteins do not share sequence homology with the middle region of bacterial ScpAs (Fig. 4 ▸ and Supplementary Fig. S1), we tested whether they may interact with the sole ScpB homologue GACE_1479 in *G. acetivorans* or Metli_0606 in *M. liminatans*. In both (His)_10_ pull-down and size-exclusion chromatographic analyses, the ScpA and ScpB proteins derived from these two species did not interact with each other, indicating that their longer middle region does not support ScpB binding (Fig. 5 ▸).

Together, these analyses demonstrate that archaeal ScpAs generally lack the ScpB-binding sequence found in bacterial ScpAs and thus they are unable to interact with ScpB.

### 3.6. Archaeal ScpA interacts with the Smc neck   

**Figure 6:**
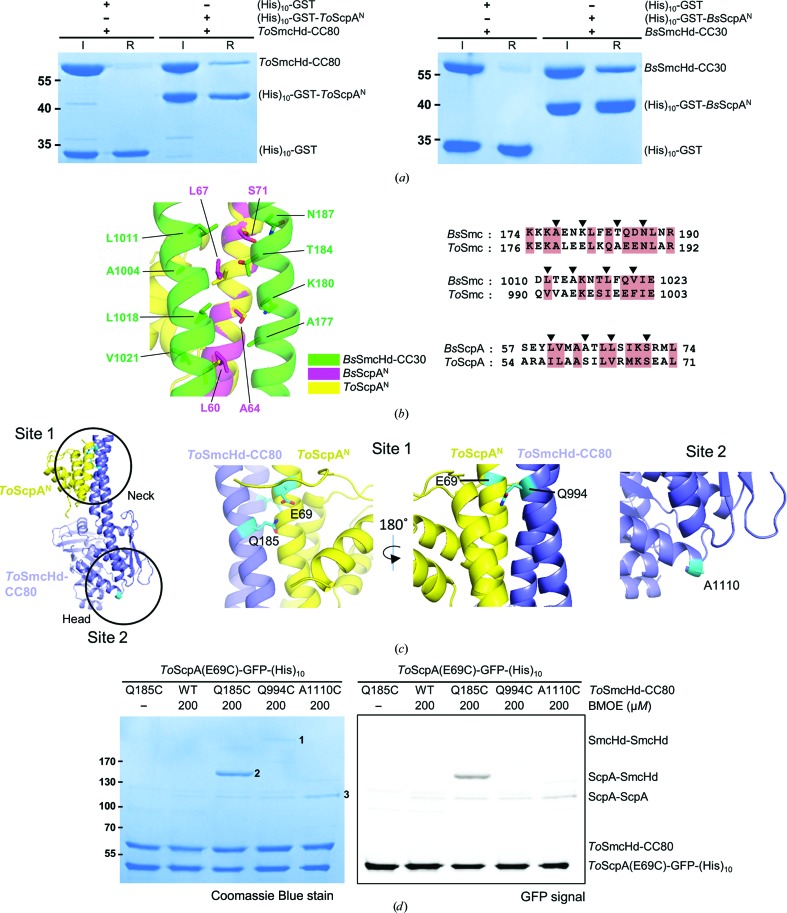
Interaction between ScpA^N^ and the Smc neck derived from *T. onnurineus.* (*a*) Pull-down assays using the indicated proteins and Ni–NTA resin. (His)_10_-GST-*To*ScpA^N^ and (His)_10_-GST-*Bs*ScpA^N^ were incubated with *To*SmcHd-CC80 and *Bs*SmcHd-CC30, respectively, in a 1:1 molar ratio (20 µ*M*) and were applied to Ni–NTA resin. Proteins retained on the resin after washing with 30 m*M* imidazole solution were visualized on an SDS–polyacrylamide gel. (*b*) Modeling the interaction between *To*ScpA^N^ and the *To*Smc neck. *To*ScpA^N^ was structurally aligned with *Bs*ScpA^N^ bound to *Bs*SmcHd-CC30 (PDB entry 3zgx; Bürmann *et al.*, 2013 ▸). Sequence alignments were performed using *Clustal Omega* (Sievers *et al.*, 2011 ▸). The black arrows indicate the residues involved in interaction at the binding interface between *Bs*ScpA^N^ and *Bs*SmcHd-CC30. Manual adjustment according to the structural alignment was unnecessary. (*c*) Cysteine positions introduced for BMOE cross-linking experiment. Based on the model of *To*ScpA–*To*SmcHd-CC80 (left), Glu69 on *To*ScpA and Gln185, Gln994 and Ala1110 on *To*SmcHd-CC80 were selected as the mutation sites (right three panels; cyan sticks). (*d*) BMOE cross-linking. The cross-linked bands are labeled from 1 to 3, and the identity of these bands were deduced based on the GFP signal (right) and mass spectrometry (Supplementary Table S3).

The N- and C-terminal regions of archaeal ScpAs are conserved throughout archaea and exhibit high sequence homology to those of bacterial ScpAs. Previously, the cWHD of *P. furiosus* ScpA was shown to interact tightly with the head domain of *P. furiosus* Smc (Diebold-Durand *et al.*, 2017 ▸; Bürmann *et al.*, 2013 ▸), as similarly observed for the bacterial counterparts (Kamada *et al.*, 2017 ▸; Diebold-Durand *et al.*, 2017 ▸). We asked whether the N-terminal domain of archaeal ScpA interacts with the Smc neck, a head-proximal region of the coiled coil, as was observed for bacterial ScpA (Bürmann *et al.*, 2013 ▸) and similarly for the kleisin subunit of yeast cohesin (Gligoris *et al.*, 2014 ▸) and condensin (Hassler *et al.*, 2019 ▸). *To*ScpA^N^ with a (His)_10_-GST tag and a *To*Smc head domain with an 80-residue coiled-coil stretch (*To*SmcHd-CC80) were purified and subjected to a (His)_10_ pull-down assay. For a control experiment, (His)_10_-GST-tagged *B. subtilis* ScpA^N^ (*Bs*ScpA^N^) and *B. subtilis* Smc (*Bs*Smc) head domain with a 30-residue coiled-coil stretch (*Bs*SmcHd-CC30) were purified. A fraction of *To*SmcHd-CC80 was pulled down by (His)_10_-GST-*To*ScpA^N^, indicating an interaction, albeit weak, between the two proteins [Fig. 6 ▸(*a*)]. In comparison, a more robust pull-down of *Bs*SmcHd-CC30 was observed [Fig. 6 ▸(*a*)], which is consistent with the high-affinity interaction observed with ScpA^N^ and SmcHd-CC30 from *G. stearo­thermophilus* (Kamada *et al.*, 2017 ▸). We next probed whether *To*ScpA^N^ also interacts with the neck of *To*Smc by thiol-specific bismaleimidoethane (BMOE) cross-linking. We prepared three mutant pairs of *To*ScpA and *To*SmcHd-CC80, both of which contained a single cysteine substitution, based on a structure-based sequence alignment of *To*ScpA with *Bs*ScpA and of *To*SmcHd-CC80 with *Bs*SmcHd-CC30 [Fig. 6 ▸(*b*)]. The three mutant pairs contain a common E69C mutation on *To*ScpA and either a Q185C, a Q994C or an A1110C mutation on *To*SmcHd-CC80. The Q185C and Q994C mutations are located on the Smc neck, whereas the A1110C mutation is located in the head domain [Fig. 6 ▸(*c*)]. In the ScpA^N^–Smc neck interface of the aligned structure, the E69C–Q185C pair (inter-C^α^ distance of 7.6 Å) appeared to be cross-linkable, while the E69C–Q185C pair (inter-C^α^ distance of 13.0 Å) did not, considering the length of BMOE. The E69C–A1110C pair (inter-C^α^ distance of 68.9 Å) was selected as a negative control. Cross-linking of these mutant pairs by BMOE resulted in one outstanding and several weak cross-linked protein bands on a denaturing polyacrylamide gel [Fig. 6 ▸(*d*)]. The use of (His)_10_-GFP-fused *To*ScpA [*To*ScpA-GFP-(His)_10_] was important for identification of the cross-linked bands, which was further confirmed by mass spectrometry (Supplementary Table S3). The most slowly migrating bands [band 1 in Fig. 6 ▸(*d*)] and fast migrating bands [band 3 in Fig. 6 ▸(*d*)] were identified as cross-linked species between head domains (*To*SmcHd–*To*SmcHd) and between ScpA proteins (*To*ScpA–*To*ScpA), respectively, which are likely to arise from random encounters of the exposed cysteine residues. Cross-linking between *To*ScpA-GFP-(His)_10_ and *To*SmcHd-CC80 [band 2 in Fig. 6 ▸(*d*); *To*SmcHd–*To*ScpA] was observed only in the *To*ScpA(E69C)–*To*SmcHd(Q185C) pair, which is consistent with the distance (7.6 Å) between the two cysteine positions in the structural alignment. These analyses together suggest that the interaction between ScpA^N^ and the Smc neck is conserved in *T. onnurineus* and probably in other archaeal species, although this interaction appears to be weaker than that between ScpA^N^ and the Smc neck in bacterial species.

## 4. Discussion   

In this study, we chose and purified six pairs of archaeal ScpA and ScpB homologues that reflect the variabilities in the genomic locations of *scpA* and *scpB* and in the length of the middle region in ScpA. In the source archaeal organisms, ScpB is the sole homologue of bacterial ScpB. None of the pairs exhibited a physical interaction between ScpA and the ScpB homologue. In these organisms, Smc and ScpA are likely to form a binary complex, which can be designated ‘Smc–ScpA’, lacking the kite subunit ScpB.

### 4.1. Prevalence of the kite-less Smc-based complex in archaea   

In total, only 160 out of 640 analyzed archaeal species contained both ScpA and ScpB. Of these, 148 species contain ScpAs with a middle region that is clearly shorter (30–47 residues) than that of bacterial ScpAs (61–72 residues) and they thus lack the polypeptide segment required for ScpB binding in bacteria. This segment is fairly hydrophobic and is conserved among bacterial ScpAs. The remaining 12 species, belonging to the orders Archaeoglobales or Methanomicrobiales, contain ScpAs with a middle region that is somewhat longer (49–66 residues) but lack meaningful sequence homology with the ScpB-binding segment found in bacterial ScpAs. Remarkably, all six tested archaeal ScpAs failed to interact with the sole putative ScpB partner, regardless of the genomic positions of the *scpA* and *scpB* genes or the length of the middle region in ScpA (Figs. 2 ▸ and 5 ▸). Our extensive experiments thus failed to identify a single pair of interacting ScpA and ScpB proteins. Therefore, archaeal ScpAs in general are unlikely to form a ternary complex with Smc and ScpB, and the kite-less Smc–ScpA complex appears to be prevalent in the archaeal domain of life.

### 4.2. What is the role of the kite subunit?   

The ScpB subunit in the bacterial Smc–ScpAB complex is known to be as important as Smc and ScpA in supporting normal cell growth and is essential for the recruitment of Smc to the chromosome (Minnen *et al.*, 2016 ▸). In *Gs*Smc–ScpAB, the kite subunit ScpB was shown to negatively regulate the interaction of ScpA^N^ with the Smc neck via steric hindrance (Kamada *et al.*, 2017 ▸). ScpB bound to ScpA clashes with the Smc head if ScpA^N^ (within the ScpAB subcomplex) were to simultaneously bind to the Smc neck, and this steric hindrance prevents ScpA^N^ from binding to the Smc neck. However, in contrast to this observation *in vitro*, cross-linking experiments showed that ScpA^N^ within the ScpAB subcomplex is allowed to bind to the Smc neck *in vivo*, conceivably as a result of a structural rearrangement in ScpA^N^ that removes the steric clash (Kamada *et al.*, 2017 ▸). It is unknown how such a structural rearrangement could take place and why negative regulation by ScpB is required for the molecular mechanism of Smc–ScpAB. It may be needed for the assembly of asymmetric Smc–ScpA rings rather than Smc dimers with ScpA bound to only one of the two Smc monomers. If so, then archaeal Smc dimers may be able to assemble asymmetric rings by other means or they may form a mixture of both variants with only the asymmetric ring form being functional. The kite subunits of the Smc5/6 complex have been implicated in DNA binding (Zabrady *et al.*, 2016 ▸). It is unclear whether ScpB has DNA-binding capabilities. Regardless, our results imply that DNA binding by ScpB is not essential in the archaeal Smc-based complex since it can function in the absence of kite subunits.

Thus, what would the function be of the ScpB sequence homologues that are found in archaeal species? A homology search against the PDB using the *DALI* server (Holm & Laakso, 2016 ▸) showed that PYCH_12850 aligns not only with *S. pneumoniae* ScpB (*Z*-score of 11.2, r.m.s.d. of 1.9 Å) but also with MTH313 from *Methanobacterium thermoauto­trophicum* (*Z*-score of 11.0, r.m.s.d. of 1.7 Å), which is a 146-residue DNA-binding protein belonging to the MarR family (Saridakis *et al.*, 2008 ▸). Since the WHD is not only a protein–protein interaction domain but also a key component in establishing protein–DNA interactions (Teichmann *et al.*, 2012 ▸), the function of the ScpB homologues in a large fraction of archaeal species might lie in a biological activity involving DNA. In a related manner, the genes encoding a kite subunit of the Smc5/6 complex (Nse3) have been duplicated several times and diversified in placental mammals, thus giving rise to a large family of proteins called MAGE proteins with a range of functions that are not directly related to those of Smc5/6 (Palecek & Gruber, 2015 ▸).

### 4.3. The interaction between archaeal Smc and ScpA is conserved   

The observed interaction between archaeal ScpA^N^ and the Smc neck is weak, in contrast to the tight interaction between *Py*ScpA^C^ (cWHD) and the Smc head domain, which enabled the co-purification of the two proteins (Bürmann *et al.*, 2013 ▸). A similar binding property has been observed for bacterial ScpA and Smc pairs: while *Bs*ScpA^C^ and *Bs*SmcHd could be co-purified (Diebold-Durand *et al.*, 2017 ▸), the ScpA^ΔC^–ScpB and SmcHd-CC pairs derived from *B. subtilis*, *S. pneumoniae* and *G. stearothermophilus* dissociated from each other in size-exclusion chromatography (not shown; Kamada *et al.*, 2017 ▸). Notably, in the absence of *Gs*ScpB, *Gs*ScpA^N^ interacted with *Gs*SmcHd-CC more tightly, with a dissociation constant of 0.5 µ*M* (Kamada *et al.*, 2017 ▸). Likely, the substantial difference in the binding affinities of ScpA or ScpAB for the head and for the neck of Smc might be commonly required for the function of the Smc-based complexes.

## 5. Perspective   

The presented work identifies a class of Smc-based complexes that consist of Smc and ScpA but not ScpB. The Smc–ScpA complex might be the sole Smc-based complex in a large number of archaeal organisms. Our work suggests that the kite subunit might have been recruited to the Smc–ScpA complex to form the Smc–ScpAB complex, probably to meet physiological requirements in most bacterial species. Future work is necessary to delineate the detailed mechanistic roles of the kite subunit in the Smc–ScpAB complex and to address why such a role is unnecessary for chromosome condensation and segregation in many archaeal organisms. The eukaryotic condensin and cohesin complexes do not have kite subunits but hawk proteins (HEAT proteins associated with kleisins), the ancestral proteins of which are found in Lokiarchaeota, the closest relatives of the last eukaryotic common ancestor. One hypothesis is that ancestral kite subunits in ancestral Smc–kleisin–kite complexes were replaced by hawk proteins (Wells *et al.*, 2017 ▸). Alternatively, ancestral Smc–kleisin complexes might have recruited hawk proteins.

## Supplementary Material

PDB reference: ScpB from *Pyrococcus yayanosii*, 6juv

PDB reference: N-terminal domain of ScpA from *Thermococcus onnurineus*, 6ivh

Supplementary tables and figures and python script for gene locus search. DOI: 10.1107/S2052252519016634/be5282sup1.pdf

## Figures and Tables

**Table 1:** X-ray data-collection and structure-refinement statistics Values in parentheses are for the highest resolution shell.

Crystal	*To*ScpA^N^ (SeMet)	PYCH_12850 (SeMet)
Data collection		
Space group	*P*6_1_	*P*4_1_22
*a*, *b*, *c* (Å)	77.073, 77.073, 39.942	92.300, 92.300, 111.817
α, β, γ (°)	90, 90, 120	90, 90, 90
Wavelength (Å)	0.9794	0.97941
Resolution (Å)	50–2.5 (2.54–2.50)	92.3–3.04 (3.25–3.04)
*R*_merge_ (%)	9.1 (28.8)	16.3 (38.1)
〈*I*/σ(*I*)〉	36.9 (4.2)	34.3 (20.2)
Completeness (%)	99.3 (97.9)	100 (100)
Multiplicity	12.5 (5.4)	113.1 (118.9)†
Refinement		
Resolution (Å)	38.5–2.50	92.3–3.04
Total No. of reflections	180880	3148552
No. of unique reflections	8905	9775
*R*_work_/*R*_free_ (%)	21.5/26.0	23.6/26.5
R.m.s. deviations		
Bond lengths Å)	0.011	0.002
Angles (°)	1.238	0.495
Average *B* value (Å^2^)	40.17	58.32
Ramachandran plot (%)		
Favored	92.0	94.7
Additionally allowed	8.0	5.3
Generously allowed	0	0
PDB code	6ivh	6juv

† The unusually high value is owing to the merging of four data sets that are dissimilar in the data completeness in resolution shells.

**Table 2:** Occurrence and juxtaposition of *smc*, *scpA* and putative *scpB* genes in archaea

			No. of species containing (*BLAST*)			No. of species containing (*HMMER*)			No. of species containing	
Phylum	Class	Order†	*smc*	*scpA*	*scpB*	Smc	ScpA	ScpB	*smc-scpA*	*scpA-scpB*
Euryarchaeota	Methanomicrobia	Methanocellales (3)	3	3	3	3	3	3	3	3
		Methanosarcinales (62)	62	62	62	61‡	62	62	59	59
		Methanomicrobiales (28)	28	28	3	26	27‡	3	28	3
	Halobacteria	Halobacteriales (92)	92	92	0	92	91‡	0	92	0
		Haloferacales (129)	126	126	0	124‡	126	0	126	0
		Natriabales (64)	64	64	0	64	64	0	64	0
	Methanococci	Methanococcales (22)	22	21	2	22	21	2	0	0
	Archaeoglobi	Archaeoglobales (9)	9	9	9	9	9	9	9	8
	Thermoplasmata	Thermoplasmatales (12)	12	12	12	12	12	12	12	1
		Methanomassiliicoccales (6)	6	6	6	5‡	5‡	6	6	6
	Thermococci	Thermococcales (44)	44	44	44	44	44	44	33	0
	Methanobacteria	Methanobacteriales (72)	0	0	0	0	0	0	0	0
	Methanonatronarchaeia	Methanonatronarchaeales (1)	1	1	1	1	1	1	1	1
	Methanopyri	Methanopyrales (1)	0	0	0	0	0	0	0	0
Crenarchaeota	Thermoprotei	(4 orders, 77 sp.)	0	0	0	0	0	0	0	0
Korarchaeota	(No class)	(No order, 3 sp.)	3	3	3	3	3	3	3	3
Micrarchaeota	(No class)	(No order, 1 sp.)	1	1	1	1	1	1	0	1
Thaumarchaeota	Nitrososphaeria	Nitrosocaldales (1)	1	1	1	1	1	1	0	1
		Nitrososphaerales (2)	2	2	2	2	2	2	0	2
	(No class)	Nitrosopumilales (11)	11	11	11	9‡	11	11	0	11

† Values in parentheses are the total number of species in the RefSeq assembly database.

‡ One or two fewer counts in each order compared with the count by the *BLAST* search because each gene is annotated as a pseudogene.

**Table 3:** The number of amino acids in the middle region of archaeal ScpAs

Phylum	Class	Order	Length	Reference species†
Euryarchaeota	Methano­microbia	Methanocellales	36	*Methanocella arvoryzae*
		Methanosarcinales	39	*Methanosarcina barkeri*
		Methanomicrobiales	49	*Methanofollis liminatans*
	Methano­cocci	Methanococcales	47	*Methanotorris formicicus*
	Archaeoglobi	Archaeoglobales	66	*Geoglobus acetivorans*
	Thermoplasmata	Thermoplasmatales	37	*Thermoplasma acidophilum*
		Methanomassiliicoccales	40	*Candidatus* Methanoplasma termitum
	Thermococci	Thermococcales	31	*Thermococcus onnurineus*
Korarchaeota	(No class)	(No order)	30	*Candidatus* Methanodesulfokores washburnensis
Thaumarchaeota	Nitrososphaera	Nitrosocaldales	40	*Candidatus* Mancarchaeum acidiphilum
		Nitrososphaerales	37	*Nitrososphaera viennensis*
	(No class)	Nitrosopumilales	33	*Candidatus* Nitrosopumilus sediminis

† These species were used to count the number of amino acids.
