# Supplementary material for: Evidence for binary Smc complexes lacking kite subunits in archaea
Source: IUCrJ. 2020 Jan 16;7(Pt 2):193–206. doi: 10.1107/S2052252519016634 (PMC7055376; doi:10.1107/S2052252519016634)
Supplement: Supplementary file 1 [file m-07-00193-sup1.pdf]

IUCrJ

Volume 7 (2020)

Supporting information for article:

Evidence for binary Smc complexes lacking kite subunits in archaea

Jae-Hyun Jeon, Han-Sol Lee, Ho-Chul Shin, Mi-Jeong Kwak, Yeon-Kil Kim, Stephan Gruber and Byung-Ha Oh

Table S1 Closest homologues of *To*Smc, *To*ScpA and *To*ScpA in the archaeal linages

Phylum	Order	Smc	ScpA	ScpB
	Methanocellales	-	-	-
	Methanosarcinales	-	-	RLG24095.1
	Methanomicrobiales	-	WP_012617431.1	CDF31225.1
	Halobacteriales	-	PSQ48810.1	-
	Haloferacales	-	WP_114448131.1	-
	Natriabales	-	-	-
	Methanococcales	-	WP_007044099.1	WP_007044001.1
Euryarchaeota	Archaeoglobales	-	WP_012939452.1	-
	Thermoplasmatales	-	WP_110641080.1	OYT50180.1
	Methanomassiliicoccales	-	OPY34124.1	-
	Thermococcales	-	-	-
	Methanobacteriales	-	-	-
	Methanonatronarchaeales	-	RZN60182.1	-
	Methanopyrales	-	-	-
Crenarchaeota		-	-	-
Korarchaeota		RLG42445.1	WP_125740284.1	PNV78162.1
Micrarchaeota		RLG21222.1	PIT84313.1	-
Thaumarchaeota		EMR73897.1	OYT66981.1	RLG03488.1

Table S2 Protein constructs generated in this study

Description	Construct	Tag/vector[a]	Tag removal	Species
*To*ScpA	Full-length	$(His)_{10}$-MBP pMAL	X	*T. onnurineus* NA1
*To*ScpA(E69C)	Full-length, E69C	GFP-$(His)_{10}$ pET22b	X	*T. onnurineus* NA1
*To*ScpA^N	residues 1-126	CPD-$(His)_{10}$ pET22b	O	*T. onnurineus* NA1
*To*ScpA^N	residues 1-126	$(His)_{10}$-GST pProEx	X	*T. onnurineus* NA1
*To*SmcHd-CC80	residues (1-254, SGGSGGS, 911-1188)	CPD-$(His)_{10}$ pET22b	O	*T. onnurineus* NA1
*To*SmcHd-CC80[b]	residues (1-254, SGGSGGS, 911-1188)	pET30a	-	*T. onnurineus* NA1
*To*SmcHd-CC80 (Q185C)[b]	residues (1-254, SGGSGGS, 911-1188), Q185C	pET30a	-	*T. onnurineus* NA1
*To*SmcHd-CC80 (Q994C)[b]	residues (1-254, SGGSGGS, 911-1188), Q994C	pET30a	-	*T. onnurineus* NA1
*To*SmcHd-CC80 (A1110C)[b]	residues (1-254, SGGSGGS, 911-1188), A1110C	pET30a	-	*T. onnurineus* NA1
TON_1955	Full-length	$(His)_{10}$-GST pProEx	O	*T. onnurineus* NA1
*Py*ScpA	Full-length	$(His)_{10}$-MBP pMAL	X	*P. yayanosii* CH1
PYCH_12850	Full-length	$(His)_{10}$-GST pProEx	O	*P. yayanosii* CH1
PYCH_12850[c]	Full-length	pET30a	-	*P. yayanosii* CH1
*Py*SmcHd-CC80	residues (1-254, SGGSGGS, 907-1177)	CPD-$(His)_{10}$ pET22b	O	*P. yayanosii* CH1
*Ms*ScpA	Full-length	$(His)_{10}$-MBP pMAL	X	*M. soehngenii* GP6
MCON_2432	Full-length	CPD-$(His)_{10}$ pET22b	O	*M. soehngenii* GP6
*Mz*ScpA	Full-length	$(His)_{10}$-MBP pMAL	X	*M. zhilinae* DSM 4017
Mzhil_1996	Full-length	CPD-$(His)_{10}$ pET22b	O	*M. zhilinae* DSM 4017
*Ga*ScpA	Full-length	$(His)_{10}$-MBP pMAL	X	*G. acetivorans* SBH6
GACE_1479	Full-length	CPD-$(His)_{10}$ pET22b	O	*G. acetivorans* SBH6
*Ml*ScpA	Full-length	$(His)_{10}$-MBP pMAL	X	*M. liminatans* DSM 4140
Metli_0606	Full-length	CPD-$(His)_{10}$ pET22b	O	*M. liminatans* DSM 4140
*Bs*ScpA^N	residues 1-124	$(His)_{10}$-GST pProEx	X	*B. subtilis* 168
*Bs*SmcHd-CC30	residues (1-219, SGGSGGS, 975-1186)	CPD-$(His)_{10}$ pET22b	O	*B. subtilis* 168

[a]CPD: Cysteine Protease Domain derived from *Vibrio Cholerae*; GFP: Green Fluorescence Protein
GST: Glutathione S-transferase; MBP: Maltose Binding Protein
[b]Each construct was expressed with *To*ScpA (E69C).
[c]The construct was co-expressed with *Py*ScpA.

Table S3 Identification of the BMOE-crosslinked proteins by mass spectrometry

**Bands**[a]	**Score**	**Identified peptides**	**Identified protein**
1	91	YAEVAMYFNNEDRGFPIDEDEVVIK	*To*Smc
		LILENPDDPFAGGLEIEAKPAGK	
		ESIEEFIEEIEGQKR	
2	73	EASENSQFIVITLR	*To*Smc
		ALTALAFVFAIQR	
	34	FWDLVFDPTPK	*To*ScpA
		AILAASILVR	
3	114	VDPWNIDIVDLTEKYIER	*To*ScpA
		REEEITPVDILLQLVQMGK	
		VEVEPLAPPLRR	

[a]Bands: band labels in Fig. 6(*d*).

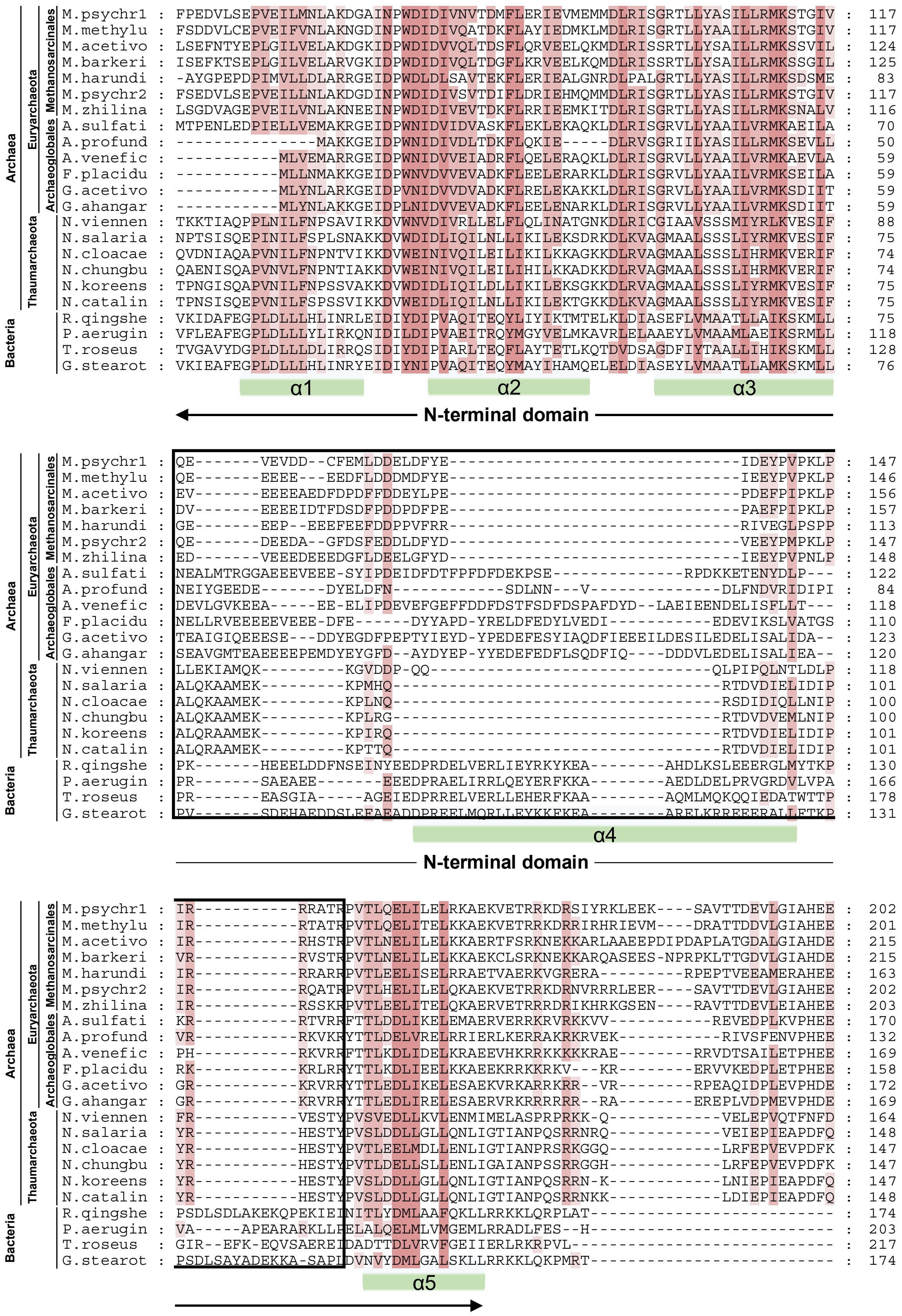
Archaea
Euryarchaeota
Methanosarcinales
Archaeoglobales
Thaumarchaeota
Bacteria
M.psychr1
M.methylu
M.acetivo
M.barkeri
M.harundi
M.psychr2
M.zhilina
A.sulfati
A.profund
A.venefic
F.placidu
G.acetivo
G.ahangar
N.viennen
N.salaria
N.cloacae
N.chungbu
N.koreens
N.catalin
R.qingshe
P.aerugin
T.roseus
G.stearot
α1
α2
α3
α4
α5
N-terminal domain
N-terminal domain

Figure S1 Multiple sequence alignment of archaeal ScpAs whose encoding gene neighbors putative *scpB*. ScpAs from 13 Euryarchaeota species and 6 Thaumarchaeota species, whose *scpA* and *scpB* are next to each other on their genome, were selected for the alignment together with 4 bacterial ScpAs. The secondary structural element of *Geobacillus stearothermophilus* ScpA$^{\Delta C}$ (*Gs*ScpA$^{\Delta C}$) is shown at the bottom of the alignment. The black box indicates the middle region of the archaeal and bacterial ScpA proteins. M.psychr1, *Methanolobus psychrotolerans* (gi: 1229239261); M.methylu, *Methanococcoides methylutens* (gi: 1175408035); M.acetivo, *Methanosarcina actetivorans* (gi: 499333721); M.barkeri, *Methanosarcina barkeri* (gi: 805410469); M.harundi, *Methanosaeta harundinacea* (gi: 973154673); M.psychr2, *Methanolobus psychrophilus* (gi: 504866340); M.zhilina, *Methanosalsum zhilinae* (gi: 503665189); A.sulfati, *Archaeoglobus sulfaticallidus* (gi: 505403278); A.profund, *Archaeogobus profundus* (gi: 502704196); A.venefic, *Archaeoglobus veneficus* (gi: 503449401); F.placidu, *Ferroglobus placidus* (gi: 288894137); G.acetivo, *Geoglobus acetivorans* (gi: 851162431); G.ahangar, *Geoglobus ahangari* (gi: 851168226); N.viennen, *Nitrososphaera viennensis* (gi: 1125725727); N.salaria, *Candidatus* Nitrosopumilus salaria (gi: 495573676); N.cloacae, *Candidatus* Nitrosotenuis cloacae (gi: 851340610); N.chungbu, *Candidatus* Nitrosotenuis chungbukensis (gi: 757131069); N.koreens, *Nitrosarchaeum koreense* (gi: 494814555); N.catalin, *Candidatus* Nitrosomarinus catalina (gi: 1198242852); P.aerugin, *Pseudomonas aeruginosa* (gi: 1440714951); R.qingshe, *Rhodococcus qingshengii* (gi: 1595910292); T.roseus, *Terriglobus roseus* (gi: 1124365071); G.stearot, *Geobacillus stearothermophilus* (gi: 1017231538).

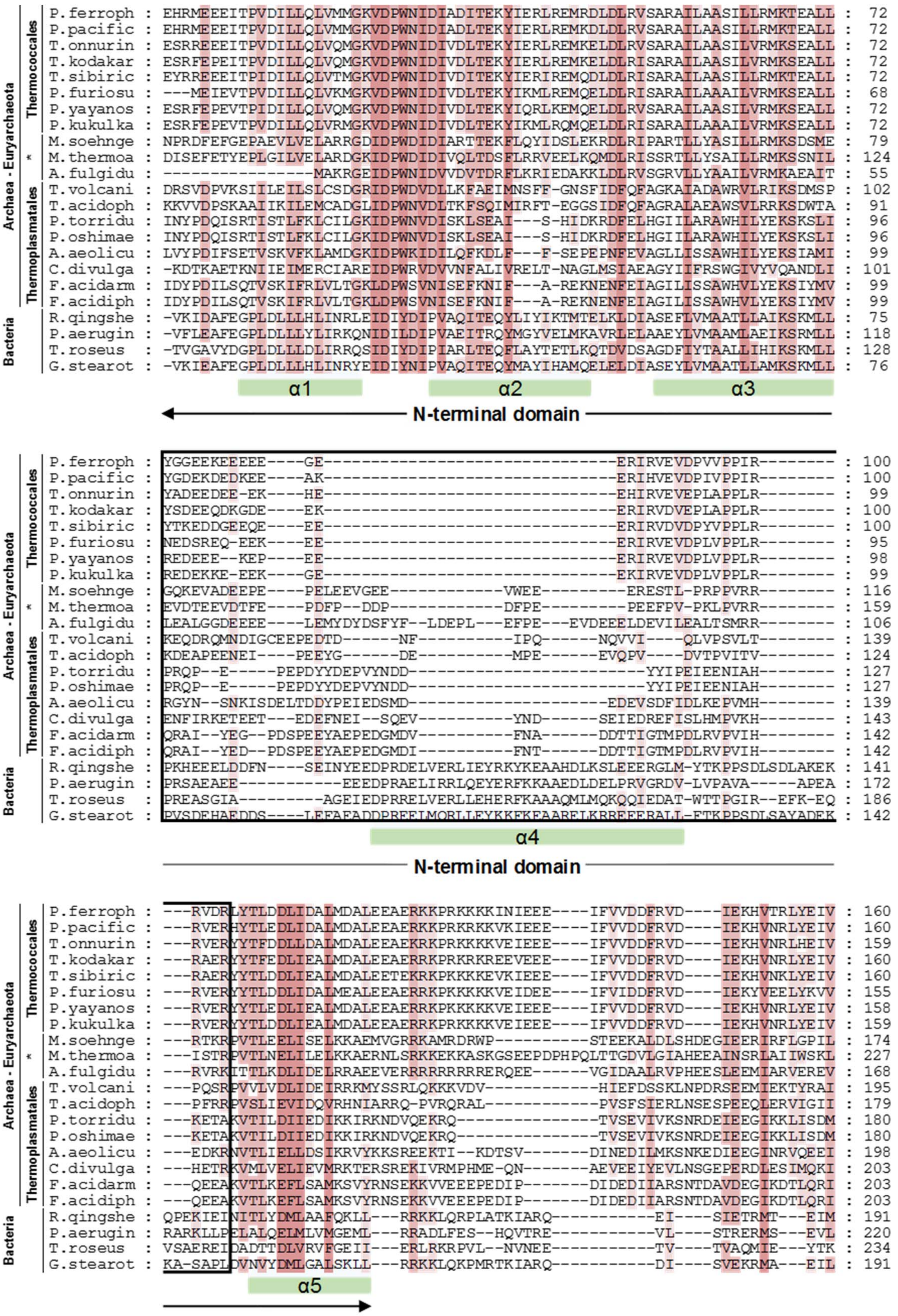

* M.soehnge and M.thermoa belong to the order Methanosarcinales and A.fulgidu belongs to the order Archaeoglobales.

Figure S2 Multiple sequence alignment of archaeal ScpAs whose encoding gene is distant from putative *scpB*. ScpAs from 19 Euryarchaeota species, whose *scpA* and *scpB* are distant from each other on their genome, were selected for the alignment together with 4 bacterial ScpAs. The secondary structural element of *Gs*ScpA$^{\Delta C}$ are shown at the bottom of the alignment. The black box indicates the middle region of the archaeal and bacterial ScpA proteins. P.ferroph, *Palaeococcus ferrophilus* (gi: 851284989); P.pacific, *Palaeococcus pacificus* (gi: 851301846); T.onnurin, *Thermococcus onnurineus* (gi: 212009177); T.kodakar, *Thermococcus kodakarensis* (gi: 499569186); T.sibiric, *Thermococcus sibiricus* (gi: 506329287); P.furiosu, *Pyrococcus furiosus* (gi: 499322490); P.yayanos, *Pyrococcus yayanosii* (gi: 337283628); P.kukulka, *Pyrococcus kukulkanii* (gi: 1056909113); M.soehnge, *Methanothrix soehngenii* (gi: 328929093); M.thermoa, *Methanothrix thermoacetophila* (gi: 116665915); A.fulgidu, *Archaeoglobus fulgidus* (gi: 2649003); T.volcani, *Thermoplasma volcanium* (gi: 499219526); T.acidoph, *Thermoplasma acidophilum* (gi: 851298378); P.torridu, *Picrophilus torridus* (gi: 48430577); P.oshima, *Picrophilus oshimae* (gi: 1174987741); A.aeolicu, *Acidiplasma aeolicum* (gi: 940338166); C.divulga, *Cuniculiplasma divulgatum* (gi: 546146250); F.acidarm, *Ferroplasma acidarmanus* (gi: 497573425); F.acidiph, *Ferroplasma acidiphilum* (gi: 1173282827); P.aerugin, *Pseudomonas aeruginosa* (gi: 1440714951); R.qingshe, *Rhodococcus qingshengii* (gi: 1595910292); T.roseus, *Terriglobus roseus* (gi: 1124365071); G.stearoth, *Geobacillus stearothermophilus* (gi: 1017231538).

S1. Python script for gene locus search

```
import os
write='OUTPUT' #output file name, can be changed into anything
writedir='OUTPUTDIR' #output directory, should be changed into the full directory path ending with
'/'
directory='INPUT' #should be changed into full directory path containing the GFF files
f=open(writedir+write+".txt",'w')
g=open(writedir+write+"_wFile.txt",'w')
filenames=os.listdir(directory)
total_tax_list=''
tax_list_A=''
tax_list_B=''
tax_list_smc=''
S_pseudo=0
A_pseudo=0
B_pseudo=0
isThereA=0
AB=0
AS=0
f.write("tax\tprev_name\tprev_locus\tcurrent_locus\tnext_name\tnext_locus\n")
def Search(tax,Prev,Current,Next):
  global tax_list_A
  global tax_list_B
  global tax_list_smc
  global isThereA
  global AB
  global AS
  global A_pseudo
  global B_pseudo
  global S_pseudo
  if 'ScpB' in Current[1] or 'segregation and condensation protein B' in Current[1]:
    if not tax in tax_list_B: tax_list_B+=tax+';'
    if 'pseudo' in Current[0]: B_pseudo+=1
  elif 'chromosome segregation protein SMC' in Current[1]:
    if not tax in tax_list_smc: tax_list_smc+=tax+';'
    if 'pseudo' in Current[0]: S_pseudo+=1
  elif 'segregation/condensation protein A' in Current[1] or 'ScpA' in Current[1]:
    if tax in tax_list_A: return
    if Prev[0]=='-': prev_locus='-'
    else: prev_locus=Prev[0].split('locus_tag=')[1].strip()
    if ';' in prev_locus: prev_locus=prev_locus.split(';')[0]
    if Prev[1]=='-': prev_name='-'
    else: prev_name=Prev[1].split('product=')[1].split(';')[0]
    current_locus=Current[0].split('locus_tag=')[1].strip()
    if ';' in current_locus: current_locus=current_locus.split(';')[0]
    if Next[0]=='-': next_locus='-'
    else: next_locus=Next[0].split('locus_tag=')[1].strip()
    if ';' in next_locus: next_locus=next_locus.split(';')[0]
    if Next[1]=='-': next_name='-'
    else: next_name=Next[1].split('product=')[1].split(';')[0]

summary=tax+'\t'+prev_name+'\t'+prev_locus+'\t'+current_locus+'\t'+next_name+'\t'+next_locus+'\n'
    f.write(summary)
    g.write(summary)
```

```
      isThereA+=1
      if 'pseudo' in Current[0]: A_pseudo+=1
      if 'ScpB' in prev_name or 'ScpB' in next_name: AB+=1
      if 'segregation and condensation protein B' in prev_name or 'segregation and condensation protein B' in next_name: AB+=1 # check if scpA,B are next to each other
      if 'chromosome segregation protein SMC' in prev_name or 'chromosome segregation protein SMC' in next_name: AS+=1 # check if smc,scpA are next to each other
      tax_list_A+=(tax+';')

for file in filenames:
  isThereA=0
  if 'gff'in file:
    db=open(directory+file,'r')
    g.write(file+"\n")
    ###get taxonomy ID
    while True:
      line=db.readline()
      if line[0:4]=="##sp":
        tax=line.split('=')[1][0:-1]
        if not tax in total_tax_list: total_tax_list+=(tax+';')
        break
    if not tax in tax_list_A:
    ###searching the three genes
      prev_locus=['-','-']
      current_locus=['-','-']
      next_locus=['-','-']
      while True:
        line1=db.readline()
        if not line1: break
        if 'RefSeq   gene' in line1 or 'RefSeq   pseudogene' in line1:
          nextline=db.readline()
          if('Protein Homology' in nextline):
            prev_locus=current_locus
            current_locus=next_locus
            next_locus=[line1,nextline]
            Search(tax,prev_locus,current_locus,next_locus)
        elif  '##' in line1:
          prev_locus=current_locus
          current_locus=next_locus
          next_locus=['-','-']
          Search(tax,prev_locus,current_locus,next_locus)
          prev_locus=['-','-']
          current_locus=['-','-']
    else: g.write(tax+'\n')
  else: continue
  if isThereA==0: g.write(tax+'\n') ### representing tax ID only, if scpA gene is not found in the file
total_tax_list=total_tax_list[0:-1]
f.write('\n# of species: '+str(len(total_tax_list.split(';')))+'\n')
g.write('\n# of species: '+str(len(total_tax_list.split(';')))+'\n')
f.write('# of species with smc: '+str(len(list(set(tax_list_smc.split(';')[0:-1]))))+'\n# of species with scpA: '+str(len(list(set(tax_list_A.split(';')[0:-1]))))+'\n# of species with scpB: '+str(len(list(set(tax_list_B.split(';')[0:-1]))))+'\n')
g.write('# of species with smc: '+str(len(list(set(tax_list_smc.split(';')[0:-1]))))+'\n# of species with scpA: '+str(len(list(set(tax_list_A.split(';')[0:-1]))))+'\n# of species with scpB: '+str(len(list(set(tax_list_B.split(';')[0:-1]))))+'\n')
```

```
f.write('\n# of species with scpA-smc neighbor: '+str(AS)+'\n# of species with scpA-scpB neighbor: 
'+str(AB)+'\n')
g.write('\n# of species with scpA-smc neighbor: '+str(AS)+'\n# of species with scpA-scpB neighbor: 
'+str(AB)+'\n')
f.write('# of species with pseudogene: scpA - '+str(A_pseudo)+'\tscpB - '+str(B_pseudo)+'\tsmc - 
'+str(S_pseudo)+'\n')
g.write('# of species with pseudogene: scpA - '+str(A_pseudo)+'\tscpB - '+str(B_pseudo)+'\tsmc - 
'+str(S_pseudo)+'\n')
### searching species which do not contain scpA,scpB or smc gene
### the results are represented as taxonomy IDs
f.write('\nscpA outlier\n')
g.write('\nscpA outlier\n')
for sp in total_tax_list.split(';'):
  if not sp in tax_list_A:
    f.write(sp+'\n')
    g.write(sp+'\n')
if total_tax_list==tax_list_A[0:-1]:
  f.write('none\n')
  g.write('none\n')
f.write('scpB outlier\n')
g.write('scpB outlier\n')
for sp in total_tax_list.split(';'):
  if not sp in tax_list_B:
    f.write(sp+'\n')
    g.write(sp+'\n')
if total_tax_list==tax_list_B[0:-1]:
  f.write('none\n')
  g.write('none\n')
f.write('smc outlier\n')
g.write('smc outlier\n')
for sp in total_tax_list.split(';'):
  if not sp in tax_list_smc:
    f.write(sp+'\n')
    g.write(sp+'\n')
if total_tax_list==tax_list_smc[0:-1]:
  f.write('none\n')
  g.write('none\n')
f.close()
g.close()
```
